# Postoperative pain after haemorrhoidal disease treatment: a still unsolved problem

**DOI:** 10.1007/s10151-023-02889-w

**Published:** 2023-12-10

**Authors:** G. Gallo, M. Goglia, M. Trompetto

**Affiliations:** 1 Department of Surgery, Sapienza University of Rome, Rome, Italy; 2 General Surgery Units, Department of Medical and Surgical Sciences and Translational Medicine, St. Andrea University Hospital, Sapienza University of Rome, Rome, Italy; 3 Department of Colorectal Surgery, S. Rita Clinic, Vercelli, Italy

Dear Sir,

Haemorrhoidal disease (HD) is a common proctological disorder with a major impact on quality of life. Surgical excision is the gold standard approach to manage grade III and IV HD but postoperative pain is a frequent complaint [1]. Indeed, the main and most common drivers of postoperative pain after conventional excisional haemorrhoidectomy (i.e. Milligan-Morgan or Ferguson) are the incorporation of sensitive mucosa and strands of the internal anal sphincter (IAS) in haemorrhoid pedicle ligatures and the denuding the anal canal of its epithelium with a consequent spasm of the IAS, the presence of tubes and packs, the swelling of the wound, the occurrence of an anal fistula or fissure, and the infection of the wound. In our opinion, the spasm of the IAS is the main driver. In fact, excisional haemorrhoidectomy causes linear wounds that extend up to the anorectal ring in the vascular pedicle and resemble an anal fissure with pain and the presence of internal sphincter hypertonia.

Several pharmacological strategies for managing postoperative pain have been described in the literature [1]. A recent meta-analysis reported that sphincterotomy is effective in terms of postoperative pain reduction, but carries a double risk of premature incontinence compared to haemorrhoidectomy alone (*p* = 0.0035), while routine use of botulinum toxin, even if effective in the first postoperative days in terms of pain reduction (< 0.001 at day 1 and 2, and *p* = 0.009 at day 7), may not be cost-effective for pain relief alone because of its high costs. On the other hand, pain was reported to be consistently and significantly reduced after topical glyceryl trinitrate administration up to 7 days after the operation (*p* = 0.012) [2].

Calcium channel blockers, such as nifedipine, have emerged in recent years owing to their effectiveness among the conservative approaches, but also in the postoperative period, especially after excisional anal surgery. Interestingly, although topical application of an ointment in patients who recently had surgery, particularly anal and perianal surgery, is usually not easy for the patient, Perrotti et al. reported that an ointment containing 0.3% nifedipine and 1.5% lidocaine may provide better pain control than 1.5% lidocaine alone at 6 h and 7 days after in patients who underwent open excisional haemorrhoidectomy (*p* < 0.011 and *p* < 0.054, respectively) [2].

Calcium channel blockers act by inhibiting the flow of Ca^2+^ into the sarcoplasm of the smooth muscle, therefore promoting sphincter relaxation. As already mentioned, decrease of the internal sphincter spasm plays a role in pain relief when combined with topical analgesia. Moreover, nifedipine has also been shown to have anti-inflammatory properties as well as the ability to modulate the microcirculation leading to the decrease of the inflammation-related sphincter hypertonicity. Interestingly, the ointment appears to act locally on the IAS, with very limited detectable systemic absorption and with no relevant local or systemic adverse events, arrhythmias or significant ECG changes reported [3].

It has been recently demonstrated that thrombosis of the mucocutaneous bridges is one of the main reasons for postoperative pain. Mesoglycan, a natural glycosaminoglycan compound (47.5% heparan sulfate, 35.5% dermatan sulfate, 8.5% chondroitin sulfate and 8.5% electrophoretically slow-moving heparin), has been proposed as an antithrombotic, profibrinolytic, anti-inflammatory and antioedema agent. It exerts its antithrombotic activity through heparan and dermatan sulfate, which are thrombin inhibitors acting through complementary pathways, antithrombin III and heparin cofactor II, respectively. The profibrinolytic activity of mesoglycan is explained by the activation of annexin A2, which favours the cleavage of plasminogen by tissue plasminogen activator causing the release of the active form of plasmin able to cleave the fibrin.

Both antithrombotic and profibrinolytic activities are carried out by reducing the concentration of plasma fibrinogen without affecting prothrombin time, partial thromboplastin time and antithrombin III levels. In fact, it has been shown to reduce pericapillary connective tissue oedema and capillary venule dilation in patients with primary venous insufficiency improving local conditions like arterial wall elasticity, transcutaneous oxygen perfusion and blood flow without any alteration of the coagulation parameters. Moreover, mesoglycan has been shown to reduce the levels of metalloproteinase-2 and metalloproteinase-9, tumour necrosis factor-α, soluble vascular cell adhesion protein 1, soluble intercellular adhesion molecule 1, and interleukin-6. A recent study demonstrated that patients who received mesoglycan postoperatively showed a significantly reduced rate of postoperative thrombosis (2.1% vs 11.1%) (*p* < 0.001) resulting in a consistent and rapid return to work 7–10 days after surgery (*p* < 0.001), compared to the control group, which represents a relevant benefit of this treatment from a socioeconomic point of view [4]. These results have been confirmed by a further multicentre study, including a much larger number of patients (192 for the control group, and 206 for the mesoglycan group) which reported a significant reduction in postoperative thrombosis among the mesoglycan-treated group at 1 and 3 weeks after surgery compared with the control group (6.3% vs 12.5% *p* < 0.05 and 3.3% vs 10.4% *p* < 0.005, respectively) [5].

Although the mechanism underlying postoperative pain is extremely complex and still not completely understood, the simultaneous use of topical application of 0.3% nifedipine and 1.5% lidocaine and the systemic administration of mesoglycan undoubtedly plays a key role, which will have to be clarified in future studies with a high level of evidence. Of course, the combination of analgesics, local ointments promoting re-epithelialization, and stool softeners is also necessary to improve the patient’s quality of life in the short term (Fig. 1).

**Fig. 1:**
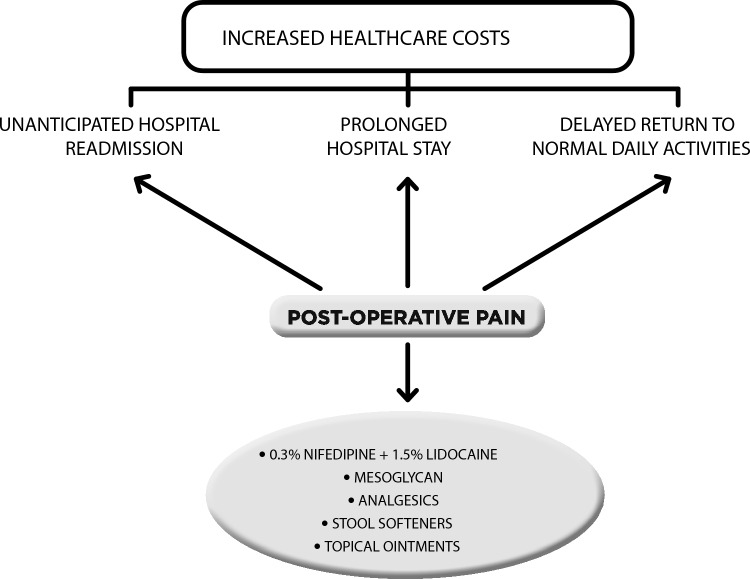
Consequences and potential treatment algorithm for postoperative pain management in patients with haemorrhoidal disease

## Data Availability

Data sharing not applicable to this article as no datasets were generated or analyzed during the current study.
